# Human Memories Can Be Linked by Temporal Proximity

**DOI:** 10.3389/fnhum.2019.00315

**Published:** 2019-09-13

**Authors:** Benjamin D. Yetton, Denise J. Cai, Victor I. Spoormaker, Alcino J. Silva, Sara C. Mednick

**Affiliations:** ^1^Department of Cognitive Sciences, University of California, Irvine, Irvine, CA, United States; ^2^Icahn School of Medicine at Mount Sinai, New York, NY, United States; ^3^Max Planck Institute of Psychiatry, Munich, Germany; ^4^Department of Neurobiology, Department of Psychology, Department of Psychiatry and Biobehavioral Sciences, Integrative Center for Learning and Memory, Brain Research Institute, University of California, Los Angeles, Los Angeles, CA, United States

**Keywords:** memory linking, neuronal ensemble, fear conditioning, temporal proximity, memory updating

## Abstract

Real-world memories involve the integration of multiple events across time, yet the mechanisms underlying this integration is unknown. Recent rodent studies show that distinct memories encoded within a few hours, but not several days, share a common neural ensemble, and a common fate whereby later fear conditioning can transfer from one memory to the other. Here, we tested if distinct memories could be linked by temporal proximity in humans. 74 young adults encoded two memories (A and B) close (3-h) or far apart (7-day) in time. One day after encoding the second memory (B), Memory A was updated by pairing it with electric shock (i.e., fear conditioning). We tested whether the memory and fear associated with Memory B would be stronger in the 3-h, compared with the 7-day condition. Results were generally consistent with rodent studies, where we found heightened Memory B fear expression when the two memories were encoded close, but not far apart, in time. Furthermore, there was less forgetting of Memory B in the 3-h compared to 7-day condition. Our results suggest that temporally proximal memories may be linked, such that updating one experience updates the other.

## Introduction

Memories encoded close in time tend to be recalled together: for example, recall of an event during your last road trip tends to facilitate the recall of other events during that holiday. In this respect, recalled memories are experienced as interconnected moments bound within a graded temporal window. These individual episodic memory traces, each of which contain details about the place and time of encoding, are initially dependent on the hippocampus (Frankland and Bontempi, 2005). The hippocampus, with its rapid learning rate (O’Reilly and Rudy, 2000) and sparse connectivity (Rolls, 2013) is ideally suited to encode discrete pattern-separated memory representations. This pattern separation leads to robustness from interference (O’Reilly and Rudy, 2000; Norman and O’Reilly, 2002) and high fidelity memories (Brady et al., 2008). However, while typical laboratory-based research in humans focuses on episodic memories as independent items, we aimed to investigate the interconnectedness of these memories when encoded close in time.

Recent experiments in rodents have demonstrated that temporally proximal, yet distinct memories share a common neural ensemble, and that this shared neuronal substrate can lead to the transfer of fear and a reduction in forgetting (Cai et al., 2016; Rashid et al., 2016). Specifically, two distinct context memories were initially encoded as neutral experiences, but when one of them was updated several days later by pairing the context with a foot shock to induce fear, this fear association transferred to the other neutral context. This implies that when memories are encoded close in time, they are “linked” and susceptible to updating by the reactivation and updating of temporally related memories. One neural mechanism that may contribute to memory linking is the excitability of neurons during encoding. Several studies have demonstrated that the excitability of neurons may predict whether they will be integrated into the same neural ensemble (Zhou et al., 2009; Yiu et al., 2014; Rogerson et al., 2016). Therefore, if the same population of neurons is excitable during the encoding of two distinct memories, then they will more likely be encoded into a common neural ensemble (Silva et al., 2009; Cai et al., 2016; Rashid et al., 2016). A recent finding in humans agrees with this memory-linking hypothesis, where inferential judgments between two semantically related stimuli were faster and more accurate when encoded within 30 min vs. across 24 h (Zeithamova and Preston, 2017). Using a fMRI multi-voxel pattern classifier, which was trained on a separate study to detect integration, Zeithamova and Preston (2017) were also able to show greater integration for items presented 30 min apart vs. 24 h. While this study did not show explicit neuronal overlap of the memory representations of temporally proximal events, both behavioral and fMRI results point toward a memory linking mechanism when two memories are encoded close in time.

Here, we test in humans, if two distinct memories encoded close in time are linked. Subjects encoded two sets of images (A and B) from difference categories (tools or animals). We manipulated the timing between encoding A and B such that one group encoded A and B 3-h apart, and the other 7 days apart (timing condition). In a later fear conditioning session, we reactivated and subsequently updated one of the memories (A) to be aversive (with footshock). We then tested whether recognition memory and fear associated with Memory B would be stronger in the 3-h, compared with the 7-day condition.

While the focus of the current study was to understand the effect of temporal proximity between encoding events, context is also a strong cue for linking, where memories that occur in the same place or with other shared contextual features may be recalled together (Dunsmoor and Murphy, 2015). Therefore, to limit any linkage effects due to a shared encoding context, encoding of A and B took place in two distinct contexts, separated by room color, computer background and experimenter. This left us with the question of which context to test recall: the room where A was encoded, the room where B was encoded, or something completely new? To control for context effects on recall, we tested in all three rooms: context 1 – the room A was encoded in, context 2 – the room B was encoded in, and context 3 – a novel room. As the novel room was completely new to the subject and not modulated by any context memory effects, we consider the context 3 to hold the clearest representation of memory linkage. Testing in each context also allowed us to ask the auxiliary question of whether the contextual component of a memory would moderate linking effects.

**FIGURE 1:**
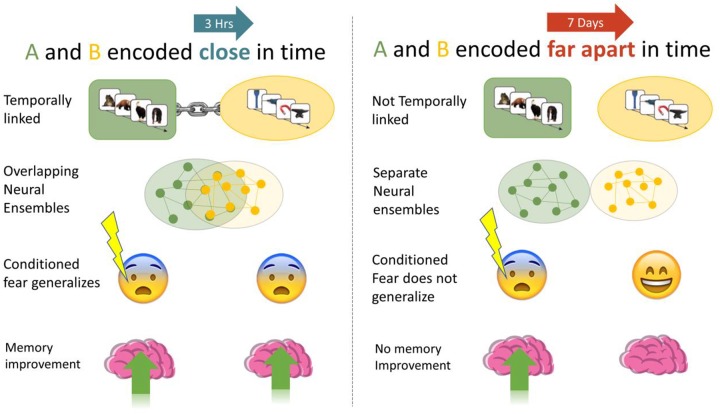
We investigated if memories encoded close in time are linked, such that manipulations of one (association of fear, memory improvement) generalized to the other. When A and B were encoded close in time (3-h) we expected higher fear response and less forgetting than when they were encoded 7 days apart.

**FIGURE 2:**
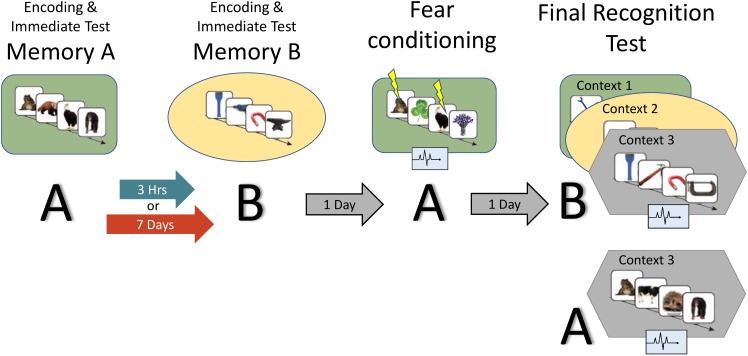
Study design: the experiment consisted of two encoding sessions, a fear conditioning session and a final recognition test session, where memory for B items as well as fear response for A and B are measured. Context is represented by the colored shapes: green rectangle = context 1, yellow ellipse = context 2, gray hexagon = context 3 (novel). A and B refer to the content being tested (animals or tools dependent on counterbalance).

Prior work has established that reactivating memories returns them to a labile state, requiring re-consolidation (Alberini and LeDoux, 2013). Labile memories are susceptible to updating, and partial reactivation of memories leads to weakening of those memories, whereas strong reactivation causes strengthening (Poppenk and Norman, 2014). We reactivated Memory A using fear conditioning, a strong reconsolidation-like process, which we predicted would lead to greater memory for the non-shocked, and temporally linked Memory B (3-h > 7 days). Memories close in time are not always related, and excessive linking of memories may be maladaptive. Thus, we predicted that linking would evince a series of small to moderate behavioral and physiological outcomes (see Figures 1, 2):

1 Behavioral Outcome: A main effect of timing condition, where memories encoded close in time (3-h) memories will be susceptible to updating, and undergo less forgetting (i.e., facilitated recall). This will be exhibited as less forgetting of memory B in the 3-h condition.
2 Psychophysiological Outcome: A main effect of timing condition where fear will generalize to memories encoded close in time. This will be exhibited as greater fear for memory B in the 3-h condition.
3 Context Outcome: A interaction of timing condition with context, where differential fear expression across context will be observed in the 3-h condition. Specifically, we expected the greatest fear in context 2 and lowest fear in context 1.

## Materials and Methods

### Participants

Seventy four Undergraduates from the University of California, Riverside took part (female = 36, mean age = 19 years) after giving informed consent in accordance with the Western Institutional Review Board and the Declaration of Helsinki. Subjects were compensated with either course credit or money for their time. All participant had at least 12 years of education, proficiency in English and regular sleep as verified by sleep diaries. Subjects were asked to abstain from alcohol and caffeine 24 h prior to each session. To control for alertness and consolidation effects, subjects were required to get at least 7 h of sleep the night before and after each session. Exclusion criteria include pregnancy, poor vision, a history of mental illness, smoking, and recent drug use.

### Paradigm

Figure 2 provides an overview of the full paradigm which took place across four sessions. At *Encoding Session 1*, subjects encoded memory A in context 1. Memory A was either a set of images of animals or tools (counterbalanced). In encoding session 2, subjects encoded Memory B in context 2. Memory B was images of the category not used in encoding session 1. Immediately following the encoding of Memory A and B, recognition memory was tested on a subset of images (*Immediate Test of A/B*). To test the temporal linkage hypothesis, the timing between Encoding Sessions 1 and 2 was randomly assigned to either 3-h (close) or 7 days (far apart) in time (*Timing Condition*, between subject’s factor). One day after Encoding Session 2, subjects experienced a *Fear Conditioning Session* where a subset of Memory A items were paired with an electric footshock in context 1 (i.e., same context as encoding; green rounded rectangle in Figure 2). During image presentation, we collected event-related physiological measures [skin conductance response (SCR), evoked heart rate (HR), and fear-potentiated startle response (FPSR)]. In the *Final Recognition Test*, which occurred one day after fear conditioning, we quantified the level of forgetting (d-prime) and physiological fear response to Memory A and B (SCR, HR, and FPSR) items. To investigate the effects of linkage on content and context independently, the Final Recognition Test of Memory B was split across three contexts (*Contexts 1, 2, and 3*; counterbalanced; within subjects factor): Context 1: the room where Memory A was encoded/shocked (green rounded rectangle in Figure 2), Context 2: the room memory B was encoded in (yellow ellipse in Figure 2), and Context 3: a novel room (gray hexagon in Figure 2). Memory A items were tested in the context 3 only. Note that the encoding-retrieval delay for Memory B is the same in both timing conditions (2 days between encoding and final test) but different for Memory A items (9 days in the 7-day condition, 2 days in the 3-h condition). We therefore do not compare the level of Memory A forgetting between timing conditions as it is confounded with the encoding-retrieval delay time.

Given the environmental reinstatement effect, whereby memory recall is facilitated when encoding and testing share the same context (Godden and Baddeley, 1975; Smith, 1979), for our fear conditioning procedure, *independent of any linkage effects* (i.e., main effect of context), we expected the strength of recall and fear of A to be facilitated in Context 1, unaffected in the Context 3, and reduced in Context 2, (Context 1 matches fear conditioning context and Context 2 matches the non-shocked “safe” context). If we observed a timing by context interaction for Memory B, where the fear in Context 2 is greater than what would be predicted by the additive effects of (i) linkage and (ii) environmental reinstatement, then this could be interpreted as the “safe” Context 2 becoming associated with fear, and therefore evidence for context linkage. The same pattern was expected for the level of forgetting of Memory B. Fear and memory associated with B items in the novel context 3 would be unaffected by any environmental-reinstatement effects or context linking effects and therefore provides the cleanest representation of memory linkage.

Because there are many factors that influence how memories are related, it was important to control the details of each context along with the semantic content of Memories A and B. For example, holding context consistent across encoding experiences would reactivate Memory A, and therefore bind Memory A and B together through context. As we wanted to maximally attribute memory linkage to time, we chose to change the context across Memory A and B encoding sessions to reduce this effect. We also implemented several other manipulations to reduce possible linking due to these confounds: first, each memory involved the encoding of different (non-semantically related) content (images of tools or animals). Second, we increased the saliency of contexts differences by changing not only the room, but wall color, computer backgrounds, and experimenter. Finally, subjects believed they were signing up for two unrelated studies and only learnt of their relation after encoding both memories.

#### Session Details

##### Encoding sessions

Fear conditioning stimuli comprised of 208 tool images and 208 animal images which were presented using MATLAB (The MathWorks Inc., 2015) and Psychtoolbox (Kleiner et al., 2007). During encoding of Memory A in Context 1, a random 108 images from one category were presented for 400 ms with 2.5 s Inter-stimulus interval (ISI) (category was counterbalanced). Subjects were asked to rate their familiarity with that item on a scale of 1–3. To account for primacy and recency effects, the first and last four images were not included in analysis. During encoding session 2, 108 images from the other category were presented. Long term image recognition memory is surprisingly accurate (Brady et al., 2008), therefore to reduce ceiling effects and maximize detection of the memory linking phenomenon, for Memory B we chose a shorter image presentation time of 250 ms (instead of 400 ms) as well as the addition of 20% Gaussian noise to each image. Encoding lasted 30 min.

Ten minutes after each encoding session, subjects began the Immediate Recognition Test (without feedback). 50 images were shown, a random 25 from the original encoding list (old) and 25 novel but of the same category (new). Participants were asked to respond if the image was new or old, and their degree of confidence (high, medium, low). No accuracy criterion was required, however, we piloted the task to 90% accuracy for the encoding of A items, and 80% accuracy for the encoding of B items. No subjects were removed due to below chance performance. Images were presented for 4 s followed by a 3 s response time and 5.5 s ISI. Participants were unaware of later memory tests (i.e., Final Recognition Test) and images presented during the Immediate Test were not shown again.

##### Fear conditioning session

Participants returned to Context 1 and viewed a random 30 images from Memory A with 80% paired with shock at image offset (CS+). None of these shocked images were tested in the immediate test. Participants were asked to respond if the image they saw was from the first half or second half of the 100 images presented during the encoding of Memory A (108 images encoded minus the eight images that control for primacy and regency). Rational for this response was to elicit reactivation of the Memory A representation, which, along with the shared encoding and conditioning context, would boost fear association with A items. Randomly intermixed with the Memory A images were 30 images from a neutral category (plants) which were not shocked, responded to, or later tested (CS−). Images were presented for 4 s, followed by a 1 s blank screen, a 3 s response time and a 4.5 s ISI. Participants were instructed that shocks may follow image presentation, and their responses did not determine the chance of shock. Physiological measures were recorded throughout and startle sound triggers were randomly paired with eight Memory A images, eight neutral images and eight ISI’s.

Electric shock was administered to the non-dominant ankle of the subject via a MATLAB triggered Biopac Mp150 and Biopac Stim 100 (Biopac Systems Inc., Goleta, CA, United States). As skin conductance varies by subject, calibration to a level deemed “unpleasant but not painful” was required (calibrated in context 1). For calibration, shock began at a barely detectable level, and was raised slowly, each time asking the subject to rate the level between 1 (barely detectable) and 10 (painful), until 8 was reached. Shocks were brief (300 ms), always less than 200 V/1A, and the calibrated level did not change throughout experiment, although habituation effects were expected.

##### Final recognition test

Participants performed a recognition memory test of Memory B items in context 1, 2, and 3 (order of contexts counterbalanced). The method followed that of Immediate Test: a random 50 images per context, with 25 old and 25 new images. No shocked or previously tested images were presented, therefore out of the 100 encoded B images, 25 were used for each of the 3 contexts at final test, and 25 during the immediate test. Images were randomly assigned to each test. Physiological measures were recorded throughout and startle sound triggers were randomly paired with eight memory A/B images and eight ISI’s. Only old images (shown only at initial encoding) were analyzed for fear measures. Stimulus timing was the same as the immediate recognition tests. Finally, we also tested 25 old and 25 new Memory A items in the Novel Context (context 3). However, there is a difference in Memory A encoding to retrieval time between timing conditions (8 days vs. 2 days), and only differences in fear expression were analyzed. The Final Recognition session lasted 90 min.

### Measures

#### Behavioral

Memory from the recognition memory task was computed using d-prime, a measure from signal detection theory used to probe the fidelity of item memory [*inverse_zscore*(hit rate)-*inverse_zscore*(false alarm rate)]. Hit rates of 1 were set to 1-1/(2N) and false alarm rates of 0 were set to 1/2N as recommended by Wixted and Lee (2019).

#### Physiological

Fear response was quantified by SCR, HR, and FPSR. Due to technical errors and equipment failure, some measures were lost for certain subjects. The *n* for each context/session is shown in Table 1 and is also reported for each analysis. All recordings were performed using a Biopac MP150 and corresponding ECG100, EMG100, and EDA100 measurement units (Biopac Systems Inc., Goleta, CA, United States). Values outside 3 standard deviations from the mean of all measurements were considered outliers, and removed.

**TABLE 1:** For each context/session.

	**Fear conditioning session**	**Final recognition test session**		
		
		**1**	**2**	**3**
SCR	64	59	61	59
HR	62	62	63	61
FPSR	51	47	50	45

*Total *n* = 74.*

##### Skin conductance response

The electrodermal response (Galvanic Skin response) measures skin conductance, a proxy of the sympathetic nervous system, psychological arousal and fear (Fowles et al., 1981). When the overall fear of a subject rises, so does the skin conductance. This process can be gradual (i.e., tonic Skin Conductance – not analyzed in current study) as well as triggered by some event (phasic SCR). Analysis of the fear induced per image was calculated in Ledalab MATLAB software (Benedek and Kaernbach, 2010) by time locking the skin conductance signal to image onset.

##### Heart rate

**FIGURE 3:**
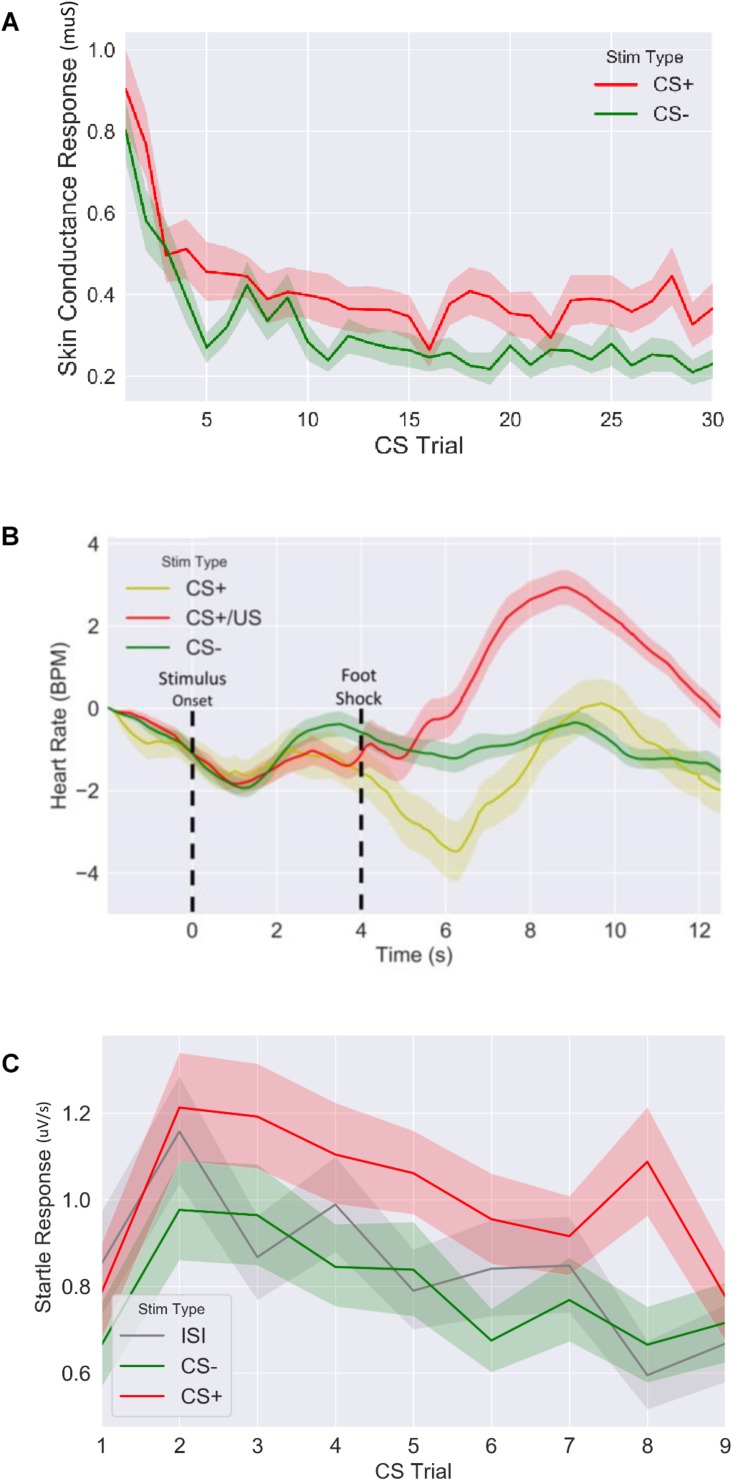
Fear response of CS+ vs. CS– trails for **(A)** skin conductance response, **(B)** evoked heart rate (as a beat per minute difference from baseline) and **(C)** fear potentiated startle response during fear acquisition session. CS: conditioned stimulus.

Heart rate is an extremely common, non-invasive measure of fear (Lazarus and Folkman, 1984). Heart rate, in average beats per minute was calculated as follows [mirroring that of Castegnetti et al. (2016)]: (1) Extract RR peaks from the Electrocardiogram signal [via Pan and Tompkins (1985) method]; (2) Take the difference between R peaks (inter beat intervals), as assign to the leading R peak time; (3) remove any inter beat intervals that are biologically implausible and likely to be artifacts (corresponding to HR of <40BPM or >160BPM); (4) Convert inter beat interval signal to BPM signal by taking the inverse and scaling; (5) Linearly interpolate this signal to get a smooth HR response for Evoked Heart Rate plots; and (6) average the HR signal across a 3–8 s window after stimulus presentation (delayed due to slow HR response to stimuli). Note that because the low sample rate of RR peaks – approx. 0.75 s/sample – and the linear spline interpolation, event triggered changes in HR may appear to occur slightly prior (∼0.25 s) to the event onset (Figure 3 exhibits this). For 80% of stimuli, shocks occurred during this 3–8 s window (at 4 s), and therefore only CS+ stimuli that do not contain shocks are used. Due to strong individual differences in heart rate, we used a difference score between each HR data point and the heart rate collected during the 5-minute session baseline (i.e., negative values represent lower heart rate, and positive values represent higher heart rate compared to baseline). While there is some discussion on the correct interpretation of event related heart rate changes, previous work consistently shows deceleration after CS+ presentation (Castegnetti et al., 2016; Sperl et al., 2016), and, as guidelines recommend (Task Force of The European Society of Cardiology, and The North American Society of Pacing, and Electrophysiology, 1996), we interpreted *lower HR as greater fear*.

##### Startle response

The human startle response is a sensitive, non-invasive measure of central nervous system activity currently used in a wide variety of research and clinical settings (see Blumenthal et al. (2005) for a technical review) whereby electromyography (EMG) is used to measure muscle activity directly under the eye. During surprise or sudden arousal these muscles twitch (often followed by a blink), and the amount of twitch gives a measure of underling psychological arousal. To induce startle, we played a brief burst of white noise for 40 ms at 104 dB, 2.5 s after image onset. The size of the startle response was quantified by (1) applying a boxcar filter to the raw EMG signal, (2) rectifying the resulting signal, and (3) Integrating the 50 ms of rectified signal following the startle sound events.

#### Analysis

Effects of memory linkage, context and their interaction on behavioral (D-prime Difference), and physiological (HR, SCR, and FPSR) dependent variables were analyzed using Mixed Linear Models using python’s statsmodels package. Mixed Linear models were preferred over Repeated Measures ANOVA as subjects missing one or more measurements are still able to contribute. Factors of the model were timing condition (3-h vs. 7-day, 7-day as the reference group) and Context (1, 2, 3, with 3 – the novel context – as reference). Context was nested within subjects and each dependent variable was entered into a separate model, with subject ID as a grouping factor. Given the strong hypotheses based off the work by Cai et al. (2016), a significant (*p* < 0.05) differences between 3-h and 7-day conditions merited further *t-*tests between timing conditions in each context separately, with a significance threshold set at *p* < 0.05.

## Results

### Fear Conditioning for Memory A

We first confirmed that our fear conditioning manipulation resulted in robust fear learning across all measures (Figure 3), where mean fear for CS+ stimuli was greater compared to mean fear for CS− averaged across the session (SCR: *t* = 4.9, *p* < 5.2E-6, *n* = 64; FPSR: *t* = 4.5, *p* < 3.9E-5, *n* = 51; HR_3__*s*__–__8__*s*_: *t* = −2.04, *p* = 0.045, *n* = 62). FPSR additionally included fear measurements during neutral ISI periods. As expected, we found fear differences to be driven by CS+ potentiation, rather than a reduction of CS− fear: CS+ demonstrated heightened fear response compared to ISI (*t* = 3.67, *p* = 6.0E-4, *n* = 52), while CS− was not different from ISI (*t* = −1.5, *p* = 0.13, *n* = 51). For later analysis of FPSR, we considered the difference between CS+ and ISI. Evoked HR followed previously reported patterns (Castegnetti et al., 2016), with a heart rate *decrease* for CS+ items compared to CS− items appearing approximately 2–3 s after image onset and remaining low for 5 s. A sharp increase in HR after the aversive shock was observed in the CS+/US condition. Given the slow HR response, and the influence of shock on HR, further analysis of HR considers the 3–8 s period following image onset for CS+ without shock pairings. No differences we observed in the level of fear conditioning between timing conditions (SCR: *t* = −0.79, *p* = 0.43, FPSR: *t* = −1.6, *p* = 0.11, HR: *t* = −0.07, *p* = 0.94) and correlations between fear measures were all non-significant (all *p* > 0.058) in the fear condition session.

### Behavioral Results

We first tested that the encoding strength did not significantly differ between the 3-h and 7-day conditions. We ran mixed model with immediate memory performance as the DV and memory list (A or B) and timing condition (3-h or 7 days) as the IV. Subject ID was used as the grouping variable. We found no significant difference between timing conditions (*p* = 0.19), and no interaction (*p* = 0.53). However, a significant difference in memory list was observed (*p* < 0.001), where A items had greater d-prime than B items. This finding was expected due to the shorter and noisier presentation of B items. A lack of encoding difference between conditions was further confirmed with independent *t*-tests between timing conditions for both immediate B memories and immediate A memories (B memories: mean d-prime_3–h_ = 1.14 ± 0.37, mean d-prime_7–day_ = 1.20 ± 46, *t* = 0.61, *p* = 0.54; A memories: mean d-prime_3–h_ = 1.53 ± 0.5, mean d-prime_7–day_ = 1.52 ± 34, *t* = 0.09, *p* = 0.93).

**FIGURE 4:**
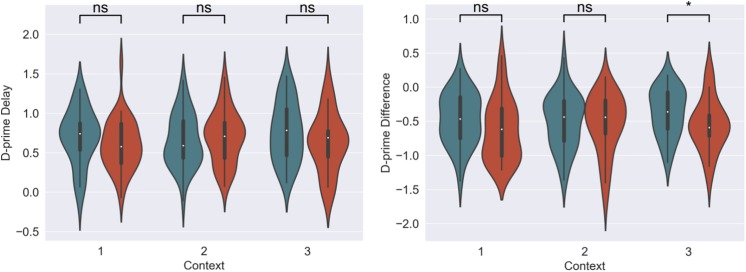
D-prime differences (final recognition test – immediate test) between 3-h and 7-day timing conditions at final test across contexts. Significantly higher memory in 3-h condition in the novel room, but not in the shock or encoding room. ^∗^Statistically significant at the 0.05 level. ns: non-significant at the 0.05 level.

For our content linkage hypothesis, we expected to see less forgetting for Memory B items at final recognition test in the 3-h condition, compared with the 7-day condition. We ran 2 mixed linear model regressions, with d-prime at final test, and a d-prime difference (final recognition test – immediate recognition test) as the dependent variables, and factors of timing condition (3-h vs. 7-day, 7-day as the reference) and context (Table 2). Context was nested within subject and dummy coded such that the novel Context 3 was the reference group. No significant effects were found when comparing d-prime at final test (all *p* > 0.115, Figure 4). However, when using d-prime difference we found a significant main effect of timing condition (Coef. = 0.19, *p* = 0.047), but no main effect of context or interaction. Follow up *t*-test revealed less forgetting (d-prime difference) in the 3-h condition compared to the 7-day condition in context 3 with a medium effect sizes, but no effect in the Context 1 or 2 (Figure 4, Context 3: *t* = 2.15, *p* = 0.035, Cohen’s *d* = 0.50; Context 1: *t* = 1.6, *p* = 0.11, Cohen’s *d* = 0.38; Context 2: *t* = 0.40, *p* = 0.68, Cohen’s *d* = −0.10; *n*_3–h_ = 36, *n*_7–day_ = 38).

**TABLE 2:** Mixed model for D-prime differences across timing condition and context.

**Parameter [dummy code reference]**	**Coef.**	**Std error**	***z***	***p***	**CI 0.025%**	**CI 0.975%**
Intercept	–0.562	0.066	–8.53	0	–0.691	–0.433
Timing condition [7-day = 0]	0.187	0.094	1.984	0.047^∗^	0.002	0.372
Context 1 [context 3 = 0]	–0.062	0.065	–0.956	0.339	–0.189	0.065
Context 2 [context 3 = 0]	0.026	0.066	0.39	0.697	–0.103	0.154
Timing condition^∗^context 1	–0.02	0.093	–0.217	0.828	–0.203	0.162
Timing condition^∗^context 2	–0.143	0.093	–1.53	0.126	–0.326	0.04

*^∗^Significant (<0.05), CI: Confidence interval around coefficient.*

### Physiological Results

**FIGURE 5:**
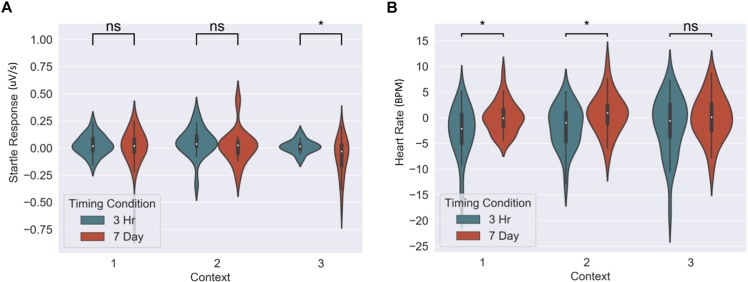
Group differences between 3-h and 7-day timing conditions at final test for **(A)** heart rate and **(B)** fear potentiated startle response. Significantly greater fear in Context 1 and 2 for heart rate and in the novel Context 3 for startle response. ^∗^Statistically significant at the 0.05 level. ns: non-significant at the 0.05 level.

The memory linking hypothesis (Silva et al., 2009) predicted that the fear associated with Memory A items during fear conditioning would also become associated with Memory B items in the 3-h condition, but not in the 7-day condition (Hypothesis #2). This was expected to manifest as higher fear for B items in the 3-h condition compared to 7-day condition during the final recognition test. We ran the same mixed level model as in the behavioral analysis for each fear measure separately (Figure 5 and Table 3). A significant main effect of timing condition was observed for FPSR (coef. = 0.085, *p* = 0.043), with no significant differences in the effects of timing condition between context (no interaction effects). However, we did note significantly reduced fear expression in Context 2, potentially driven by its association as a non-shocked “safe context” after fear condition (coef. = 0.081, *p* = 0.037). Follow up *t*-tests revealed significantly greater FPSR in the 3-h condition in Context 3 (Figure 4, Context 3: *t* = 2.19, *p* = 0.033, Cohen’s *d* = 0.63, *n*_3–h_ = 23, *n*_7–day_ = 25; Context 1: *t* = 1.65, *p* = 0.10, Cohen’s *d* = 0.49, *n*_3–h_ = 21, *n*_7–day_ = 25; Context 2: *t* = −0.25, *p* = 0.80, Cohen’s *d* = −0.08, *n*_3–h_ = 20, *n*_7–day_ = 25). For HR, no significant differences were found using our mixed model, however, *t*-tests within each context highlighted significantly greater fear for the 3-h condition in Context 1 and 2 (Context 3 *t* = −1.14, *p* = 0.26, Cohen’s *d* = −0.29, *n*_3–h_ = 29, *n*_7–day_ = 33; Context 2: *t* = −2.3, *p* = 0.023, Cohen’s *d* = −0.61, *n*_3–h_ = 26, *n*_7–day_ = 33; Context 1: *t* = −2.6, *p* = 0.012, Cohen’s *d* = −0.67, *n*_3–h_ = 28, *n*_7–day_ = 32). All SCR differences were non-significant (all *p* > 0.11). We found no difference in fear associated with Memory A items at final recognition test (FPSR: *t* = 0.61, *p* = 0.54, *n*_3–h_ = 20, *n*_7–day_ = 26; Cohens *d* = 0.18; HR: *t* = −1.36, *p* = 0.18, *n*_3–h_ = 26, *n*_7–day_ = 31, Cohen’s *d* = −0.36; SCR: *t* = −0.82, *p* = 0.42, *n*_3–h_ = 27, *n*_7–day_ = 28, Cohens *d* = 0.22).

**TABLE 3:** Mixed models for fear variables across timing condition and context.

**Parameter [dummy code reference]**	**Coef.**	**Std error**	***z***	***p***	**CI 0.025%**	**CI 0.975%**
**A: FPSR**						
Intercept	–0.047	0.029	–1.616	0.106	–0.104	0.01
Timing condition [7-day = 0]	0.085	0.042	2.022	0.043^∗^	0.003	0.167
Context 1 [context 3 = 0]	0.002	0.039	0.039	0.969	–0.075	0.078
Context 2 [context 3 = 0]	0.081	0.039	2.087	0.037^∗^	0.005	0.157
Timing condition^∗^context 1	–0.011	0.057	–0.2	0.842	–0.123	0.1
Timing condition^∗^context 2	–0.096	0.057	–1.683	0.092	–0.208	0.016
**B: Heart rate**						
Intercept	–0.014	0.78	–0.018	0.986	–1.543	1.515
Timing condition [7-day = 0]	–1.51	1.13	–1.335	0.182	–3.725	0.706
Context 1 [context 3 = 0]	0.164	0.608	0.271	0.787	–1.027	1.356
Context 2 [context 3 = 0]	0.733	0.601	1.219	0.223	–0.446	1.911
Timing condition^∗^context 1	–1.073	0.902	–1.19	0.234	–2.841	0.694
Timing condition^∗^context 2	–1.335	0.906	–1.474	0.141	–3.11	0.44
**C: FPSR**						
Intercept	0.366	0.045	8.046	0	0.276	0.455
Timing condition [7-day = 0]	–0.056	0.064	–0.88	0.379	–0.182	0.069
Context 1 [context 3 = 0]	–0.055	0.035	–1.589	0.112	–0.123	0.013
Context 2 [context 3 = 0]	–0.014	0.035	–0.399	0.69	–0.083	0.055
Timing condition^∗^context 1	0.024	0.05	0.477	0.633	–0.074	0.122
Timing condition^∗^context 2	–0.05	0.05	–0.988	0.323	–0.148	0.049

*^∗^Significant (<0.05), CI, Confidence interval around coefficient.*

**FIGURE 6:**
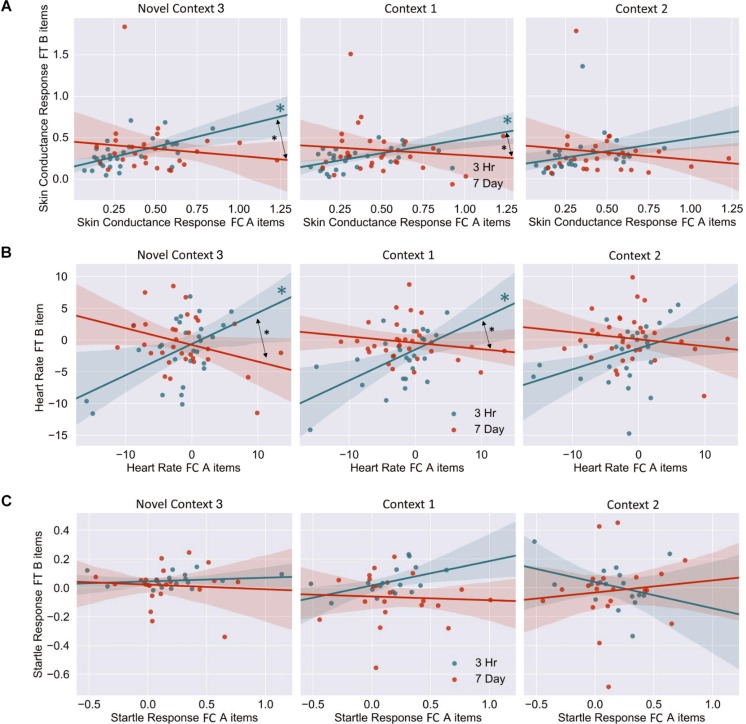
**(A)** Skin conductance response [muS], **(B)** heart rate [BPM], **(C)** startle response [uV/s], for memory A during fear conditioning correlated with skin conductance of Memory B at final test, across context. Note that when removing the potential skin conductance response outliers (i.e., those >1.25), significance remained for all but the difference between correlations in the novel room. FC, Fear Conditioning Session; FT, Final Test Session.

The mixed nature of these results across context and fear variable prompted further analysis of the linkage effect. If memories encoded close in time are linked, then we would expect a relationship between the fear associated with Memory A during fear conditioning and the fear response of Memory B during final test. That is, when memories are encoded close in time, the greater the fear associated with A during fear conditioning means the greater the amount of fear associated with B, and therefore a positive correlation between the two. To test this, we ran a mixed model for each fear variable (HR, SCR, and FPSR, Table 4). Each model tested fear during the final test session as the DV, and predictors were timing condition (7-day condition coded as 0) and memory A fear during fear conditioning (i.e., the average fear expressed during presentation of CS+ Memory A items during the fear conditioning session). Due to our lack of strong hypotheses regarding the pattern of effects between contexts, we added in fear from all 3 contexts to this model without including context as a predictor. To show a linkage effect, we expected a positive relation between the conditioned fear of memory A items, and the expressed fear of memory B items, but only in the 3-h condition (i.e., a significant interaction term but no significant term for conditioned fear of memory A items). We found a significant positive interaction coefficient for both heart rate (Coef. = 0.5, *p* = 0.003) and skin conductance (Coef. = 0.55, *p* = 0.046), but not for startle response (coef. = 0.175, *p* = 0.51). Interestingly, skin conductance showed significantly lower fear in the 3-h condition (coef. = −0.27, *p* = 0.033). This suggests that the component of SCR at final test that is not attributable to the fear conditioning session was greater in the 7-day condition, and may help explain our lack of linkage findings in the previous non-correlational analysis. We then turned to a correlational analysis within each room to confirm the mixed model findings. Specifically, for each fear measure, timing condition and room, we separately correlated the fear conditioned for memory A items during fear conditioning, and the fear expressed for memory B items during final test. In line with the linkage hypothesis, SCR and HR both exhibited sizable positive correlations in the 3-h condition, but no significant correlations in the 7-day condition (Figure 6 and Table 5). Significant differences between the correlation coefficient in the 3-h and 7-day condition were found in Context 3 and Context 1, and a similar (but weaker) pattern exists in Context 2 as well. The difference between these correlations (*z*-test after Fisher r-to-z transform) was significant in Context 3 and Context 1 for both measures. FPSR did not show any significant effects.

**TABLE 4:** Mixed models relating fear variables at final test to fear at fear conditioning across timing condition.

**Parameter [dummy code reference]**	**Coef.**	**Std error**	***z***	***p***	**CI 0.025%**	**CI 0.975%**
**A: Heart rate**						
Intercept	–0.081	0.677	–0.12	0.904	–1.408	1.246
Timing condition [7-day = 0]	–0.173	0.969	–0.179	0.858	–2.073	1.727
Memory A fear during FC	–0.13	0.115	–1.128	0.259	–0.356	0.096
Timing condition^∗^ memory A fear during FC	0.543	0.182	2.988	0.003^∗^	0.187	0.899
**B: SCR**						
Intercept	0.42	0.095	4.408	0	0.233	0.607
Timing condition [7-day = 0]	–0.27	0.127	–2.129	0.033^∗^	–0.519	–0.022
Memory A fear during FC	–0.151	0.176	–0.858	0.391	–0.496	0.194
Timing condition^∗^ memory A fear during FC	0.551	0.277	1.991	0.046^∗^	0.009	1.093
**C: FPSR**						
Intercept	0.043	0.023	1.843	0.065	–0.003	0.089
Timing condition [7-day = 0]	0.005	0.036	0.128	0.898	–0.065	0.075
Memory A fear during FC	–0.029	0.092	–0.316	0.752	–0.209	0.151
Timing condition^∗^ memory A fear during FC	0.175	0.263	0.666	0.506	–0.341	0.692

*^∗^Significant (<0.05), CI, Confidence interval around coefficient.*

**TABLE 5:** Fear measure correlations, A items at fear conditioning vs. B items at final recognition test.

**SCR**	**3 Hour**			**7-Day**			**Difference**	
			
	***r***	***p***	***n***	***r***	***p***	***n***	***z***	***p***
Context 3	**0.52**	**0.005**	**27**	–0.08	0.70	24	**2.2**	**0.02**
Context 1	**0.50**	**0.01**	**25**	–0.19	0.38	24	**2.43**	**0.02**
Context 2	0.27	0.19	25	–0.17	0.43	23	1.45	0.15
**HR**								
Context 3	**0.49**	**0.01**	**26**	–0.34	0.07	30	**3.11**	**0.002**
Context 1	**0.53**	**0.005**	**25**	–0.20	0.29	29	**2.74**	**0.006**
Context 2	0.28	0.17	24	–0.22	0.27	28	1.73	0.08
**FPSR**								
(CS+ – ISI)								
Context 3	0.01	0.97	17	–0.10	0.68	19	0.3	0.76
Context 1	0.49	0.08	14	–0.08	0.74	19	2.74	0.12
Context 2	−0.32	0.25	15	0.11	0.66	18	−1.11	0.27

*Bold values highlight significance at the 0.05 level.*

## Discussion

Retrieval of an episodic memory has been famously likened to “mental time travel” (Tulving, 1985), but how does the brain encode temporally proximal events such that time can be used as memory’s compass? In this study in humans, we tested if, similar to rodents, time could be the primary linking features between two independent memories, such that changing one memory would have a mild but detectable effect on the other memory (Silva et al., 2009). In our experiment, we aimed to limit any context overlap between two memory events, such that linking effects could be maximally attributed to temporal proximity between the two encoding sessions. Thus, subjects memorized a set of images from a specific category (Memory A) in a specific context, and either 3-h or 7 days later, returned to a different context (different room, room color, study name and experimenter) to encode another list of images from a different semantic category (Memory B). In a fear conditioning session, we paired electric shock with Memory A, thereby associating fear with A items. We hypothesized that if memories are temporally linked, manipulation of Memory A should affect physiological response to B, similar to rodent freezing response. In addition, we predicted that linking would affect the level of forgetting of linked memories through a reconsolidation-like mechanism. When tested in a final recognition testing session, we found less forgetting and greater fear for the non-shocked B items, as well as stronger positive correlations between the fear associated with the shocked A items and the non-shocked B items, but only when these memories were encoded close in time.

There are some unanswered questions and limitations with the current study. We found differential patterns of results across behavioral and physiological fear measures, where some measures fell in line with the content linkage hypothesis (memory effects, FPSR fear differences, SCR and HR correlations), while others (HR and SCR fear, FPSR correlations), which are purported to quantify a similar phenomenon, showed no effect (although no results actively refuted the linkage hypothesis). Likewise, results across context were mixed: results for the level of forgetting and fear were the most consistent in the novel Context 3 (e.g., level of forgetting, FPSR differences, SCR and HR correlations). However, we observed no memory effects in Context 1 or 2, which may be due to uncontrolled contributions of interference or environmental reinstatement. Similarly, for fear, a lack of robust context^∗^timing condition interactions argued against any modulatory effect of context linkage on temporal proximity. Further, the saliency of context 1 may lead to better memory in the 3-h condition compared to the 7-day condition due to the difference in delay between encoding and test in this context (2 days in the 3-h condition, vs. 9 days in the 7-day condition). With the paradigm used, we cannot rule out that the lack of a memory difference in Context 1 was due to stronger recall of the discrete encoding event during encoding session 1 (Memory A), and thus not the linked encoding event (Memory B), although the alternate viewpoint is also defendable, where higher context 1 recall improves recall of memory A and hence memory B through linkage. This discrepancy places further precedence on the novel context 3 as the purest representation of linkage, and again highlights the need for more work investigating the effect of context. In general, the study is limited by the small effect size observed coupled with a relatively small sampled size. Therefore, conclusions should be considered tentative and further work with sufficient control conditions is required to solidify the presence of temporal memory integration on the scale of hours in humans.

What neural processes lead to memory linkage? Our work builds on several other studies in rodents (Zhou et al., 2009; Rashid et al., 2016) where neuronal overlap lead to memory linkage. In a similar paradigm to ours, Cai et al. (2016) introduced mice to three different contexts (A, B, C). A and C were separated by a week, while B and C were within the same day. Context C was later paired with shock. When tested 1 day later, fear response in the non-shocked B context was the same as in the shocked context C, and both were significantly higher than A. Calcium imaging of CA1 hippocampal neurons exhibited greater similarity in neural representations for B and C (encoded within a day) compared to A and C (encoded a week apart). As previously proposed (Silva et al., 2009) when mice were again placed back in context B, recalling the memory of context B triggered the recall of context C and led to the mice freezing in context B, despite never being shocked in that context. While we do not have direct evidence to show that the linking observed in our human subjects was due to neuronal overlap between Memory A and B, other studies using pattern analysis techniques in fMRI and ECog suggest that memory linking is mediated by overlapping neural representations (Manning et al., 2011; Schlichting et al., 2015). Similar techniques should be employed in future studies to shed light on this critical temporal aspect human memory.

Open questions remain. For example, the temporal order of memories may play a role. While Cai et al. (2016) encoded two context memories, and associated fear with the second, we encoded two contexts and paired fear with the first. In an ecologically motivated hypothesis, the retrieval of an aversive context memory during re-exposure to a prior, predictive, and temporally linked context is adaptive and serves to warn of negative stimulus to come [i.e., as in Cai et al. (2016)]. However, reinstatement of a fearful memory during re-exposure to a memory that occurred after the negative context may be less adaptive (initial encoding after the fearful context, therefore less predictive). It remains to be seen if the reinstatement of linked memories via neuronal overlap is bi-directional. Along with fear, our experiment showed an reduction in the forgetting of episodic memory as quantified by d-prime, but the mechanism by which this occurred is still unknown. A tentative explanation is that new events are integrated with previous, similar event memories at the time of encoding (Shohamy and Wagner, 2008), and this process can modify the original memory. This modification may transfer to the linked (second) memory through their shared neuronal ensemble. For example, during the fear conditioning session, reactivation of Memory A, and its subsequent pairing with shock may causes an increase in Memory A strength, and the neurons that encode it. Neural allocation theory predicts that this upregulated Memory A ensemble overlaps with Memory B ensemble, and hence Memory B is also strengthened (Silva et al., 2009). Unfortunately, without a no-shock control condition the fear conditioning facilitation of Memory A cannot be quantified as it is confounded with the forgetting time and further work is needed.

The amount of time between memories may also play a role in understanding mechanisms of memory linking, as differing mechanisms have been proposed when events occur closer or further apart in time (Morton et al., 2017). Similar to “place cells” for spatial memories, temporal integration on the order of seconds may be organized by “time cells” in the hippocampus (MacDonald et al., 2011; Kraus et al., 2013; Morton et al., 2017). As with neural excitability in the memory linking hypothesis (Silva et al., 2009), time cells may help bias the encoding of new information such that sequential events occupy overlapping neuronal representations. For integration of memories at longer time scales, neurogenesis in the dentate gyrus may be important (Aimone et al., 2006), although there is some controversy over the presence of neurogenesis in humans (see Bergmann et al., 2015 for review). The interaction of these three processes: time cells, neural excitability, and neurogenesis in the integration of memories along a continual dimension of time, from seconds to weeks, is unknown in the current study, and a promising avenue of future research. Further, it remains to be seen whether time alone is sufficient to abolish temporal linkage or whether other active processes, such as sleep, may be necessary.

Although sleep is well known for its role in memory consolidation, especially for events encoded during the previous day (Diekelmann and Born, 2010; Mednick et al., 2013), it is also possible that sleep may play a role in resetting linkage representations across days. Perhaps the temporal integration of memories, or lack thereof, is not due to time per say, but is instead conditional on a period of sleep occurring between memory events. Certainly, sleep appears to refresh the brain’s episodic encoding capacity for new knowledge (Mander et al., 2011) and a tentative hypothesis is sleep resets neuronal excitability and therefore restricts temporal-linking across neuronal ensembles. Along with our study, the study by Zeithamova and Preston (2017) is consistent with this intriguing idea: for two object memories learnt on the same day vs. across sleep, an inference that required both memories was faster and more accurate when they were encoded on the same day, suggesting more integration between memories. However, no study is yet to dissociate the role of time vs. sleep the in memory linking phenomenon, and more work is needed.

Memory-linking is particularly important in the context of memory disorders. Without effective memory-linking, the processes that promote normal memory retrieval might result in memory failure, such as failing to link “caution” to a previously on, and still hot, stove might cause someone to burn themselves. On the other hand, an overabundance of memory-linking can also be maladaptive. One example of this is post-traumatic stress disorder (PTSD), a debilitating disorder characterized by re-experiencing a traumatic event in the form of persistent and intrusive memories. In PTSD, the traumatic memory may be triggered by initially neutral experiences that become over-linked and generalize to the traumatic experience. Fear generalization has most often been studied with respect to similarity across physical dimensions (Hayes et al., 2012) (e.g., visual cues). In contrast, very little focus has been placed upon similarity across temporal dimension. This lack of research is surprising given the fundamental role that time has in memory formation/utilization: to make sense of the content of a memory it is important to consider both the sensory features that comprise a memory, as well as the procession of these features with respect to the passage of time. Discerning how the brain links (or separates) memories across time may lead to novel PTSD treatments. In summary, convergent behavioral and physiological markers show a possible linking between two separate memories that were encoded within a short temporal window. Further work is needed to investigate the underling mechanisms of integration as well as how they may be influenced by circadian processes and sleep, and their role in clinical disorders involving emotional memories.

## Data Availability

The datasets generated for this study are available on request to the corresponding author.

## Ethics Statement

The studies involving human participants were reviewed and approved by the Western Institutional Review Board. The patients/participants provided their written informed consent to participate in this study.

## Author Contributions

BY: ideation, study design, literature review, data collection, data analysis, and manuscript writing. DC: ideation, study design, literature review, and manuscript editing. VS: recommendations for study design and data analysis. AS: ideation. SM: ideation, study design, and manuscript editing.

## Conflict of Interest Statement

The authors declare that the research was conducted in the absence of any commercial or financial relationships that could be construed as a potential conflict of interest.
